# Time units for learning involving maintenance of system-wide cFos expression in neuronal assemblies

**DOI:** 10.1038/s41467-018-06516-3

**Published:** 2018-10-08

**Authors:** Ananya Chowdhury, Pico Caroni

**Affiliations:** 1 Friedrich Miescher Institut, Maulbeerstrasse 66, CH-4058 Basel, Switzerland; 2 Present Address: Depts. of Neurobiology, Psychology, Psychiatry and Biobehavioral Sciences; Integrative Center for Learning and Memory; Brain Research Institute, UCLA, Los Angeles, CA, 90095 USA

**Keywords:** Cellular neuroscience, Consolidation

## Abstract

Repeated experiences may be integrated in succession during a learning process, or they may be combined as a whole within dedicated time windows to possibly promote quality control. Here we show that in Pavlovian, incremental and incidental learning, related information acquired within time windows of 5 h is combined to determine what mice learn. Trials required for learning had to occur within 5 h, when learning-related shared cues could produce association and interference. Upon acquisition, cFos expression was elevated during 5 h throughout specific system-wide neuronal assemblies. Time window function depended on network activity and cFos expression. Local cFos activity was required for distant assembly recruitment through network activity and distant BDNF. Activation of learning-related cFos assemblies was sufficient and necessary for time window function. Therefore, learning processes consist of dedicated 5 h time windows (time units for learning), involving maintenance of system-wide neuronal assemblies through network activity and cFos expression.

## Abstract (web summary)

Learning often involves multiple exposures and trials, but it is not known whether those are treated independently, or integrated during dedicated time windows. Here, Chowdhury and Caroni show mice learn new associations during 5 h time windows, where related experiences are integrated in a process requiring coordinated cFos-activated neuronal assemblies.

## Introduction

An isolated experience can produce long-lasting memories, but learning often involves multiple interactions with related information^1–4^. The outcome of these interactions could be integrated incrementally, independent of when individual interactions occur. Alternatively, integration might occur within dedicated periods of time, breaking down learning processes into discrete time units. The latter possibility might provide brain mechanisms for content and quality control in learning, and to avoid interference through spurious observations, but whether learning processes involve dedicated time windows for associative learning has remained unclear.

Local pharmacological manipulation of dopamine D1 receptor signaling within about 5 h from acquisition modulates the strength of long-term memories^5^, and neuronal assemblies accounting for memories can be combined within a time window of about 5 h from acquisition^6,7^. These findings suggest the existence of molecular, cellular, and network mechanisms that might support the combination of related memories in learning during a time window of about 5 h, but whether and how these proposed mechanisms might play together to determine what is learned is currently poorly understood.

Here, we investigated whether learning might involve dedicated time windows for integration of individual related experiences, and what might be the underlying molecular/cellular, circuits and systems mechanisms. We show that in spatial, motor, fear, and incidental learning, related information acquired within time windows of 5 h is combined to determine whether and what is learned. For learning to occur, sufficient trials had to be executed within 5 h, and learning-related shared partial cues produced association of otherwise unrelated information and interference with learning when occurring within 5 h. Single related but goal-contradicting trials within 5 h were sufficient to disrupt learning, and such interference could not be overcome within the same time unit. Addressing cellular and network mechanisms underlying time unit duration and function, we show that these involve maintenance of specific system-wide neuronal assemblies^8,9^ through network activity^10^, cFos expression^11,12^, and BDNF signaling^12–14^.

## Results

### A 5 h time window for learning and associative binding

**Fig. 1:**
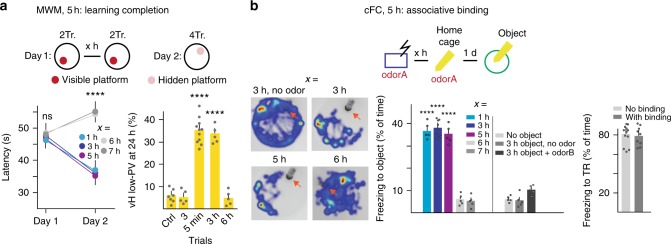
A 5 h time window for learning and associative binding. **a** Time window to complete MWM learning. Left: latencies as a function of time interval between first and second group of two trials on Day1. Two-way RM ANOVA, Interaction, *P* < 0.0001, *n* = 5, 8, 5, 5, 5; right: PV plasticity (low-PV contents in vH at 24 h; Ctrl: swim control without platform); One-way ANOVA, *P* < 0.0001, *n* = 7, 5, 11, 5, 5. Tr. trial, *x* numbers of hours as indicated in different protocols; Day2: 4 trials. **b** Associative binding of neutral object to fear memory during 5 h time unit. *x* number of hours following acquisition of cFC. Schematic: boxes of different colors indicate different contexts; object: Falcon tube. Left, center: Representative heat maps (left) and quantitative analysis (center; one-way ANOVA, *P* < 0.0001, *n* = 5, 9, 5, 5, 5, 5, 5, 5) of novel context exploration in the presence of object (Falcon tube; orange arrows). Right: Fear memory binding (3 h after acquisition) does not influence fear response to conditioning context (tested at 26 h; unpaired *t*-test, *t*(19) = 0.8568, *P* = 0.4022, ns, *n* = 12, 9). Error bars: SEM; *P* < 0.05 (*), 0.01 (**), 0.001 (***), 0.0001 (****). For a more detailed description of the statistical analysis please refer to Supplementary Table 1

To investigate possible temporal constraints in learning, we first defined numbers of individual trials that, under our experimental conditions, led to behaviorally detectable progress in Morris water maze^15^ (MWM; incremental spatial learning) or rotarod training protocols^16^ (RR; incremental motor skill learning). Under our experimental conditions, regimes of four daily trials led to detectable progress, whereas regimes of two daily trials did not (Supplementary Figure 1). We then determined whether there might be critical time windows within which those four trials needed to be completed in order to promote learning. We assessed MWM learning behaviorally as incremental improvement in daily performance^15^, and cellular-molecularly as learning-related increase in the fraction of parvalbumin (PV) neurons exhibiting low expression levels of PV (PV plasticity) in ventral hippocampus (vH)^17,18^. In MWM training protocols, the first day (Day1) involves a visible platform, whereas all subsequent days (e.g. Day2) involve a submerged (hidden) platform (Fig. 1a). We tested MWM protocols consisting of 2 + 2 (2 not sufficient, 4 sufficient) trials, separated by increasingly long time intervals (Fig. 1a). Any time interval up to 5 h produced learning (as detected on Day2) and hippocampal PV plasticity that were undistinguishable from that detected upon a conventional 5 min inter-trial interval protocol (Fig. 1a). By contrast, intervals of 6 h or more not only produced no behavioral learning, but also no learning-related PV plasticity in vH (Fig. 1a). In closely comparable 2 + 2 trials protocols, RR learning was equally effective when the total of four trials was delivered within a time window of up to 5 h, whereas time windows of 6 h or more led to no learning and no PV plasticity in primary motor cortex^18^ (M1; Supplementary Figure 2). These findings suggest that training essential for behavioral learning needs to be integrated within time units of 5 h.

The 5 h time window might reflect a time when learning-related information is most effectively integrated, which would be reminiscent of recent findings concerning the merging of memory engrams^6,7^. To investigate this notion, we placed a neutral object (Falcon tube), together with the odor associated with Pavlovian conditioning (partial cue) during a period of 30 min into the home cage of mice that had undergone contextual fear conditioning (cFC; Fig. 1b). Supporting the notion that partial cues are associated effectively to learning within time windows of 5 h, mice exposed on the next day to a novel (i.e. neutral) context with the object, but without the odor, avoided the object and froze only if object + partial cue presentation had occurred within 5 h after cFC (Fig. 1b). This associative binding to fear memory occurred 5 h but not 7 h after acquisition regardless of whether the initial cFC was carried out at 4 a.m., 10 a.m., 4 p.m. or 10 p.m. (Supplementary Figure 3), suggesting that the duration of the time window for associative binding was not affected by the circadian rhythm. In control experiments, the associative binding protocol, which led to freezing values of 35–40% of the time in a new context with the object, did not influence the magnitude of freezing responses to the conditioning context, which were about 80% of the time regardless of whether mice had learned the additional association between object and fear (Fig. 1b).

### Shared partial cues within 5 h time units modify learning

**Fig. 2:**
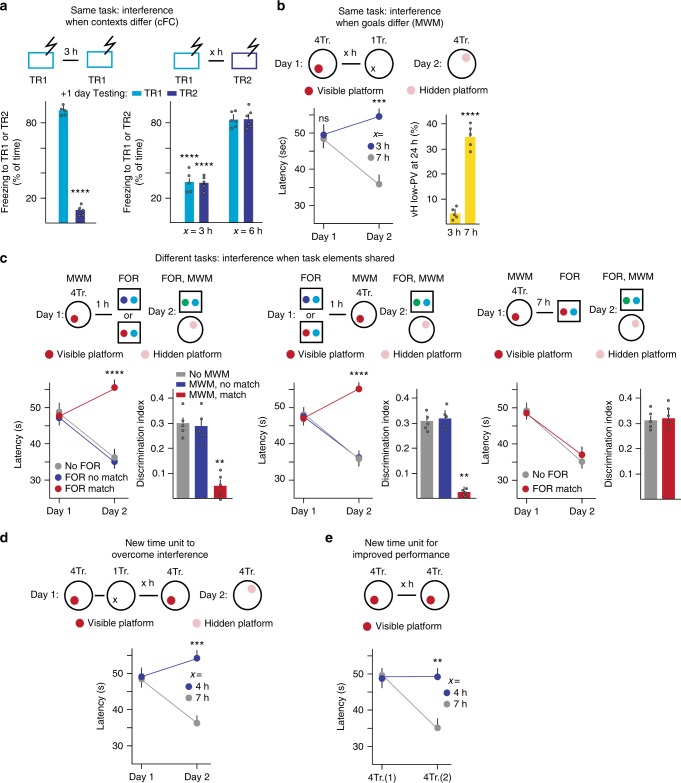
Interference through shared partial cues within 5 h time units. **a** Specificity of freezing response in the contexts, when shocked in TR1 twice (unpaired *t*-test, *t*(8) = 30.14, *P* < 0.0001), *n* = 5, 5). Interference when cFC in TR1 is followed within 5 h time unit by cFC in different context TR2. Freezing at recall 1 day after acquisition assessed in TR1 or TR2 (two-way ANOVA, *P* < 0.0001, *n* = 5, 5, 6, 6). **b** Interference in MWM learning by single trial without platform carried out during 5 h time window. Left: latencies [two-way RM ANOVA, Interaction *P* = 0.0140, *n* = 5, 5; right: PV plasticity (unpaired *t*-test, *t*(8) = 9.272, *P* < 0.0001, *n* = 5, 5). **c** Interference between different tasks (MWM learning and object recognition memory (FOR)) carried out during same 5 h (but not 7 h) time unit only if they share one task-related element (red: platform same as one object; Latency, MWM first (left) [two-way RM ANOVA, Interaction *P* = 0.0044, *n* = 5, 5, 5]; FOR: (One-way ANOVA, *P* = 0.0007, *n* = 5,5,5; Latency, FOR first (center) [two-way RM ANOVA, Interaction, *P* = 0.0098; *n* = 5, 5, 5]. FOR: (one-way ANOVA, *P* = 0.0009, *n* = 5, 5, 5). **d** Overcoming interference by single trial without platform in MWM learning through further four trials with platform not achieved within same learning unit [two-way RM ANOVA, Interaction *P* = 0.0043, *n* = 5, 5]. **e** Behaviorally detectable improved performance in MWM learning detected upon initiation of new learning unit [two-way RM ANOVA, Interaction *P* = 0.0233, *n* = 5, 5]. Error bars: SEM; *P* < 0.05 (*), 0.01 (**), 0.001 (***), 0.0001 (****). For a more detailed description of the statistical analysis please refer to Supplementary Table 2

We then explored experimental conditions under which learning of distinct but behaviorally related content within time units of 5 h might produce interference and disruption of learning. We first investigated delivery of two cFC protocols (same type of learning) in different contexts (conflicting conditioned stimulus associations). cFC in a first context (TR1), and then again in a different context (TR2) 3 h later, led to a dramatic reduction of freezing (35% instead of 80% of the time) to TR1 or TR2 on the next day (Fig. 2a). By contrast, no interference with freezing was detected when the two conditioning contexts separated by 3 h were identical, or when cFC in TR1 and TR2 were separated by 6 h (Fig. 2a). These results suggest that when the two cFC protocols in different contexts are carried out within 3 h from another, none of the two contexts is associated with a robust fear memory, possibly because detection of the US in TR2 conflicted with association of the US with TR1.

We then investigated the possible impact of a single trial without escape platform (same task; closely comparable behavior but no escape goal) for MWM learning. Remarkably, when four trials required for learning (visible platform) were completed within 25 min (conventional 5 min inter-trial interval), a single trial without platform delivered at 3 h, but not at 7 h was sufficient to suppress any learning-related improved performance (Day2, hidden platform) and learning-related PV plasticity (Fig. 2b). Therefore, and like the two cFC protocols in different contexts, these results suggest that when the association between water maze setting and goal (the escape platform) is contradicted within a 5 h time window, mice do not associate the maze setting with the escape platform, and do not learn to navigate searching for the hidden platform.

Finally, we investigated whether interference might also occur among entirely distinct learning tasks, in cases where we provide a task-relevant element that is shared between the two tasks. First, we found that when an object recognition memory task (incidental learning; familiar object recognition (FOR)^18^) and MWM training were carried out in the absence of shared elements within the same 5 h time unit, the two tasks did not interfere with each other (Fig. 2c). Notably, however, when one of the objects during acquisition of FOR closely resembled the escape platform (for these particular experiments FOR object and MWM escape platform were the same), learning of FOR and MWM were both disrupted as detected by performance on the next day (Fig. 2c). The outcome of these experiments was not influenced by whether MWM was carried out before or after FOR (Fig. 2c). Furthermore, when MWM and FOR were carried out with a shared element but with a 7 h delay from each other, no interference was detected (Fig. 2c). Taken together, these results provide evidence that, within the 5 h time unit, contrasting task-related goals among trials belonging to the same or closely related tasks, or individual shared task-related goals between different tasks are sufficient to profoundly disrupt learning.

In a further set of behavioral experiments to define features of time units for learning, we investigated the possibility that initiation of a new time unit might be necessary to overcome interference in learning. Indeed, in MWM learning, four additional trials with visible platform carried out within the same time unit for learning failed to overcome the impact of a single trial without escape platform as detected on Day2 (hidden platform; Fig. 2d). By contrast, when the additional four trials were carried out after the end of the time unit (7 h), they produced robust learning undistinguishable to carrying out a single unperturbed MWM learning unit (Fig. 2d). Finally, when a second set of four trials (visible platform) was carried out within the same time unit, it was not associated with improved performance, whereas the improved performance was comparable to that detected on a subsequent training day when the second set of four trials (visible platform) were carried out 7 h after the first one (Fig. 2e). Therefore, overcoming associational interference or producing improved performance as a consequence of MWM learning requires initiation of a new time unit.

### Five-hour network activity and cFos for time unit function

**Fig. 3:**
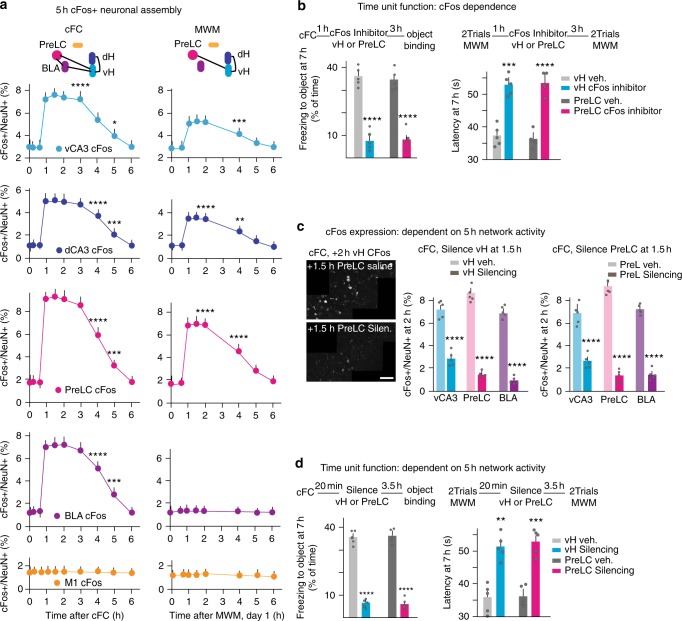
Time unit for learning dependent on 5 h network activity and cFos function. **a** Time course of cFos induction in systems involved in cFC or MWM learning (four trials). Schematics: systems analyzed involved in cFC or MWM (connected by lines). cFC [One-way ANOVA; vCA3: *P* < 0.0001, dCA3: *P* < 0.0001, PreLC; *P* < 0.0001, BLA: *P* < 0.0001, M1: *P* = 0.5472], *n* = 5; MWM [One-way ANOVA, vCA3: *P* < 0.0001, dCA3: *P* < 0.0001, PreLC: *P* < 0.0001, BLA: *P* = 0.7868, M1: *P* = 0.9978]. *n* = 5. **b** Inhibiting cFos activity 1 h after acquisition locally in vH or PreLC prevents further time unit function in cFC and MWM learning. cFos inh.: local delivery of cFos inhibitor; veh.: vehicle (cFC: two-way ANOVA, Treatment, *P* < 0.0001, *n* = 5, 5, 5, 5; MWM: two-way ANOVA, Treatment, *P* < 0.0001, *n* = 5, 5, 5, 5). **c**, **d** 5 h network activity at systems used in learning required for system-wide cFos expression (right: representative examples of vH cFos immunoreactivity with and without PreLC silencing; bar = 50 μm; vH silencing: Two-way ANOVA, Interaction, *P* = 0.0002, Treatment, *P* < 0.0001, *n* = 5 each; PreL silencing: Two-way ANOVA, Interaction, *P* = 0.0009, Treatment, *P* < 0.0001, *n* = 5 each) (**c**) and time unit function (cFC: two-way ANOVA, Treatment, *P* < 0.0001, *n* = 5 each; MWM: Two-way ANOVA, Treatment, *P* < 0.0001, *n* = 5 each) (**d**). Silen.: local and reversible silencing of brain system through pharmacogenetic PV neuron activation. Error bars: SEM; *p* < 0.05 (*), 0.01 (**), 0.001 (***), 0.0001 (****). For a more detailed description of the statistical analysis please refer to Supplementary Table 3

To investigate cellular and systems mechanisms that might underlie 5 h time units for learning, we focused on the induction of cFos expression in brain regions known to be involved in cFC^19^ or MWM learning^20^. The immediate early gene and transcription factor cFos is a reporter of learning-related plasticity in neurons^12,21,22^, and activity in assemblies of learning-related cFos+ neurons is involved in memory recall^8,9,22,23^. For cFC^19^, we focused on ventral and dorsal hippocampus (vH, dH), basolateral amygdala (BLA) and prelimbic cortex (PreLC) (primary motor cortex (M1) as negative control; i.e. not implicated in this form of learning); for MWM^20^ we focused on vH, dH and PreLC (BLA and M1 as negative controls) (Fig. 3a). Contents of cFos+/NeuN+ neurons in vH CA3 were markedly elevated 1 h after cFC (Fig. 3a; Supplementary Figure 4). Peak contents of cFos+ neurons were sustained from 1 to 3 h, cFos values were still half-maximally elevated at 4 h, and had returned to baseline cage control values at 6 h (Fig. 3a; Supplementary Figure 4). Comparable time courses of cFos induction and maintenance were detected in dH, BLA, and PreLC, whereas no cFos induction was detected in M1 (Fig. 3a). Upon MWM learning, cFos induction up to 4–5 h after training was detected in vH, dH, PreLC but not BLA (Fig. 3a).

To determine whether cFos function might be causally involved in learning unit function, we inhibited cFos activity locally in vH or PreLC 1 h after acquisition with a small-molecule compound that prevents transcriptional activity of cFos-containing AP1 complex^24^. Indeed, local treatment (Supplementary Figure 5) with the cFos inhibitor was sufficient to suppress learning unit function (cFC, freezing to object; MWM, 2 + 2 trials; Fig. 3b). In control experiments, freezing to context (as opposed to freezing to object) at 7 h was not affected by vH cFos inhibition at 1 h (Supplementary Figure 6), consistent with the notion that cFos activity was specifically required for associative learning during the time unit for learning.

These findings raised the question of what might be the mechanisms underlying cFos+ neuronal assembly maintenance throughout brain regions used for learning a task. To determine whether network activity^3,10^ might have a role in maintaining cFos expression and learning unit function throughout the 5 h time window, we carried out silencing experiments in single brain regions implicated in the studied behavior. These experiments involved activation of local parvalbumin+ (PV+) inhibitory interneurons in PV-Cre mice through ligand-induced triggering of pharmacogenetic activator (floxed PSAM-carrying AAV9 (rAAV9-CAG-flox-PSAM(Leu41Phe,Tyr116Phe)5HT3-WPRE)) delivered locally (Supplementary Figure 5) through a Cre-dependent AAV^5,25–27^. Under these experimental conditions, silencing lasts about 30 min^26^. Silencing vH any time from just after (5 min) to 4 h after acquisition of cFC led to rapid (within 15 min) and complete loss of cFos in vH, as well as throughout the memory network, including dH, BLA, and PreLC (Fig. 3c; Supplementary Fig. 7). Likewise, silencing PreLC led to loss of cFos in PreLC, vH, and BLA (Fig. 3c). Furthermore, silencing vH or PreLC 1 h after acquisition also led to network-wide loss of cFos induced upon MWM learning (Supplementary Fig. 7). In control experiments, silencing M1 did not affect vH or PreLC cFos induced upon cFC, and silencing BLA did not affect cFos induced upon MWM learning (Supplementary Fig. 7). In parallel to system-wide loss of cFos, silencing vH or PreLC at +20 min suppressed associative binding to fear memory at +4 h (measured as freezing to object at 7 h), and learning-unit function at 4 h in the 2 + 2 trials MWM training protocol (Fig. 3d). Therefore, activity in distributed networks involved in learning is required during a 5 h time window to prevent loss of cFos and of learning unit function.

### cFos-dependent extension of time unit function

**Fig. 4:**
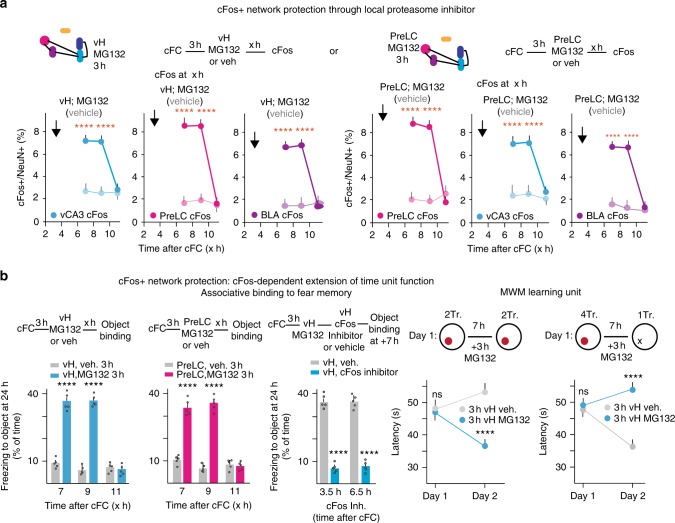
cFos-dependent extension of time unit for learning upon local inhibition of protein degradation. Preventing protein degradation locally with proteasome inhibitor is sufficient to extend time window of cFos expression throughout the cFos network. Local delivery of MG132 at 3 h to vH (left); pale colors: vehicle controls. vCA3: Two-way ANOVA, Interaction *P* < 0.0001, PreLC: Two-way ANOVA, Interaction, *P* < 0.0001, BLA: Two-way ANOVA, Interaction, *P* < 0.0001; *n* = 9, 9, 5, 6, 5, 6. Local delivery of MG132 at 3 h to PreLC (right); PreLC: Two-way ANOVA, Interaction, *P* < 0.0001, vCA3: Two-way ANOVA, Interaction, *P* < 0.0001, BLA: Two-way ANOVA, Interaction, *P* < 0.0001, *n* = 5, 5, 5, 5, 5, 5. **b** cFos-dependent extension of time unit function. Left: extension of fear memory binding time unit by local delivery of proteasome inhibitor. vH treatment: Two-way ANOVA, Interaction, *P* < 0.0001, *n* = 5 each; PreL treatment: Two-way ANOVA, Interaction, *P* < 0.0001, *n* = 5 each. Center: cFos dependence of time unit extension: Two-way ANOVA, Treatment, *P* < 0.0001, *n* = 5 each. Right: extension of time unit for trial completion [Two-way RM ANOVA, Interaction, *P* = 0.0023, *n* = 5 in each group. Extension of time unit for interference [Two-way RM ANOVA, Interaction, *P* = 0.0003, *n* = 5 in each group. Error bars: SEM; *P* < 0.05 (*), 0.01 (**), 0.001 (***), 0.0001 (****). For a more detailed description of the statistical analysis please refer to Supplementary Table 4

To further relate cFos expression in distributed neuronal assemblies to learning unit function, we sought to extend the duration of learning-induced cFos expression. Local delivery of proteasome inhibitor MG132^28,29^ to vH at +3 h produced a long-lasting extension of elevated cFos+ neuron contents in vH, with values at +7 h and at +9 h that were comparable to those at +3 h, and return to baseline values at +11 h (Fig. 4a). Notably, local delivery of MG132 to vH also effectively extended learning-related cFos expression in dH, BLA, and PreLC (Fig. 4a). Likewise, MG132 delivery to PreLC extended cFos expression in PreLC and vH (Fig. 4a).

In parallel to extension of cFos expression by local delivery of the proteasome inhibitor, the time window for learning unit function as detected by binding to fear memory or by sufficient trials for MWM learning was now extended to up to +9 h (Fig. 4b). Prevention of protein degradation with MG132 followed by local delivery of cFos inhibitor abolished binding to fear memory, indicating that the extended time window for memory binding depended on cFos expression and activity (Fig. 4b). Local interference with protein degradation also extended the time window during which a single interfering trial was sufficient to suppress MWM learning (Fig. 4b). Taken together, these results provide evidence that time units for learning can be extended in a cFos-dependent manner through local interference with protein degradation.

### cFos neuronal assembly activity mediates time unit function

**Fig. 5:**
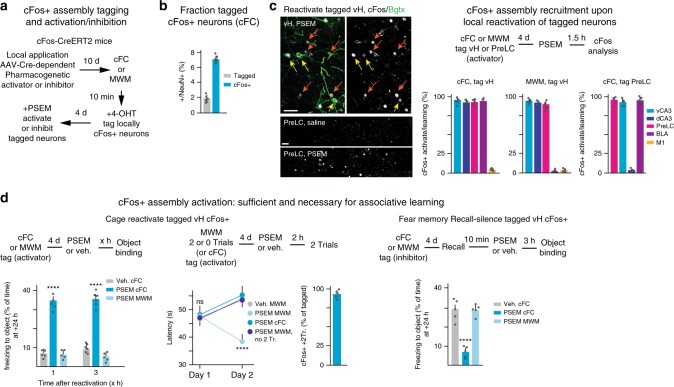
Activity in local cFos+ neuronal assemblies sufficient and necessary for time unit function. **a** Schematic of cFos+ neuron tagging and reactivation protocols. 4-OHT: Tamoxifen derivative used in these experiments. **b** Fractions of tagged and total cFos+ neurons upon cFC acquisition in vH. *N* = 6 mice each. **c** System-wide cFos+ neuronal assembly recruitment upon local reactivation of tagged cFos+ neurons. Left: representative images of vH tagged neuron re-activation experiment; yellow arrows: tagged (Bgtx+) and cFos+ neurons; orange arrows: cFos+ neurons induced by activation of tagged neurons; Right: 0 h cFos+/NeuN+ baseline values subtracted from learning values, and vehicle cFos+/NeuN+ values subtracted from PSEM activation values; bars: 40 μm. *N* = 5 mice for each group. **d** Role of cFos+ assembly re-activation and inhibition for time unit function. Left: Reactivation of cFC (but not MWM) tagged vH cFos+ neurons is sufficient for associative fear memory binding, Two-way ANOVA, Condition, *P* < 0.0001, *n* = 5, 6, 6, 6, 5, 5). MWM learning: re-activating, 4 days later (now defined as Day1), vH cFos+ neurons tagged after two trials is sufficient to replace the first two trials in MWM learning [Two-way RM ANOVA, Interaction, *P* = 0.0017, Condition, *P* = 0.0030, *n* = 5; right: fraction of vH two-trials MWM tagged neurons that are cFos+ after two more trials (*n* = 5 mice). Right: fear memory recall 4 days after acquisition reopens a time unit for memory binding, which is suppressed by inhibition of tagged cFos+ fear memory neurons (One-way ANOVA, *P* < 0.0001, *n* = 5 for each group). Error bars: SEM; *P* < 0.05 (*), 0.01 (**), 0.001 (***), 0.0001 (****). For a more detailed description of the statistical analysis please refer to Supplementary Table 5

To investigate the role of neuronal activity for time unit function specifically in learning-related cFos+ assemblies rather than generally in implicated brain regions, we carried out cFos assembly activation and inhibition experiments^8,9,22,23^. We tagged cFos+ neurons by delivery of hydroxy-Tamoxifen shortly after acquisition to activate Cre recombinase in mice expressing Tamoxifen-dependent CreERT2 in the cFos locus^30^. We combined these manipulations with previous local delivery (Supplementary Figure 5) of AAV carrying Cre-dependent pharmacogenetic activator or inhibitor channels^25^ (floxed PSAM-carrying AAV9 (activation: rAAV9-CAG-flox-PSAM(Leu41Phe,Tyr116Phe)5HT3-WPRE; inhibition: rAAV9-CBA-flox-PSAM(Leu141Phe,Tyr116Phe)GlyR-WPRE), to activate or inhibit the learning-related cFos+ neurons (Fig. 5a).

In these experiments, only a fraction of the cFos+ neurons induced upon learning were labeled and hence accessed by the tagging procedure in vH or PreLC. Thus, the fraction of Bungarotoxin+/NeuN+ (i.e. successfully tagged) neurons visualized 4 days after learning (time needed for expression in these trapping experiments) was only 16–22% of the fraction of cFos+/NeuN+ neurons induced 90 min after acquisition (i.e. at the peak of cFos induction; Fig. 5b). Pharmacogenetic reactivation of tagged vH cFos+ fear memory neurons induced robust expression of cFos (to an extent up to 80–95% of the fraction of cFos+ neurons induced upon learning) in vH, dH, PreLC and BLA, but not M1 (Fig. 5c). Likewise, reactivation of tagged PreLC cFos+ fear memory neurons induced robust expression of cFos (again up to 80–95% of the fraction of cFos+ neurons induced upon learning) in PreLC, vH, and BLA (Fig. 5c). Whether, and to what extent, the cFos+ neurons induced upon reactivation of tagged neurons are identical to those originally expressing cFos upon acquisition remains to be determined (but see Fig. 5d). However, supporting specificity in cFos induction, pharmacogenetic reactivation of tagged vH MWM cFos+ neurons induced robust expression of cFos in vH, dH, PreLC, but not BLA (Fig. 5c).

Reactivation of vH cFos+ fear memory neurons in the home cage 4 days after cFC, followed 1 or 3 h later by presentation of (object + odor) produced effective binding of fear memory to object (Fig. 5d). In control experiments, reactivation of vH MWM cFos+ memory neurons did not produce binding of fear memory to object, indicating that binding specifically involved activation of cFos+ fear memory neurons (Fig. 5d). In a second test, we tagged cFos+ neurons after two trials of MWM (Fig. 5d). Reactivation of those cFos+ MWM neurons in vH was sufficient to replace the first two trials in a new MWM time unit (Fig. 5d). Most (>90%) of the reactivated tagged cFos+ neurons were cFos+ upon two additional MWM trials (Fig. 5d). In control experiments, reactivation of cFC cFos+ neurons or omission of the subsequent two trials failed to produce MWM learning (Fig. 5d).

Finally, in related experiments addressing necessity of cFos+ neurons for associative memory binding, recall of fear memory upon placing mice in training context reopened a time window for (object + odor) associative binding, which was suppressed by pharmacogenetic silencing of tagged vH cFos+ fear memory neurons (Fig. 5d). Taken together, these results provide evidence that activity in learning-related cFos+ neuronal assemblies is sufficient and necessary for time unit function.

### Local cFos and distant BDNF for assembly recruitment

**Fig. 6:**
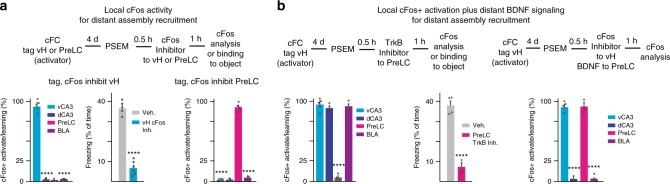
Distant assembly recruitment involving local cFos activity and distant BDNF signaling. **a** Local re-activation of tagged cFos+ neurons, combined with local inhibition of cFos activity prevents network-wide recruitment of cFos+ neurons without affecting local recruitment; values as described in Fig. 5c (local vH: one-way ANOVA, *P* < 0.0001, *n* = 5 each; local PreLC: one-way ANOVA, *P* < 0.0001, *n* = 5 each). Center: absence of network-wide cFos recruitment prevents time unit function (unpaired *t*-test, *t*(9) = 10.17, *P* < 0.0001, *n* = 5,6). **b** Role of BDNF signaling for distant cFos+ neuron recruitment. Left: local re-activation of tagged cFos+ neurons in vH, combined with local delivery of TrkB inhibitor to PreLC specifically prevents induction of cFos expression in PreLC; values as described for Fig. 5c (one-way ANOVA, *P* < 0.0001, *n* = 5 each) and time unit function (unpaired *t*-test, *t*(8) = 8.525, *P* < 0.0001, *n* = 5 each). Right: local delivery of BDNF to PreLC is sufficient to disinhibit cFos induction upon local re-activation of tagged vH cFos+ neurons, combined with local inhibition of cFos activity in vH (one-way ANOVA, *P* < 0.0001, *n* = 5 each). Error bars: SEM; *P* < 0.05 (*), 0.01 (**), 0.001 (***), 0.0001 (****). For a more detailed description of the statistical analysis please refer to Supplementary Table 6

To investigate the function of cFos protein in memory network recruitment and memory binding, we carried out cFos+ neuron tagging and reactivation experiments combined with local pharmacology experiments. Reactivation of tagged vH cFos+ fear memory neurons followed by local delivery of cFos inhibitor (block cFos transcriptional activity) to the same vH did not prevent robust induction (80–90% of the total fraction of cFos+ neurons induced upon cFC) of learning-related cFos+ neurons in vH (Fig. 6a). Notably, however, in the same experiments, local inhibition of cFos activity in vH prevented induction of cFos+ neurons in distant memory network systems, such as PreLC, dH, or BLA (Fig. 6a). In parallel, local inhibition of cFos in vH prevented re-induction of associative memory binding (Fig. 6a). Likewise, reactivation of tagged PreLC cFos+ fear memory neurons followed by local delivery of cFos inhibitor to PreLC did not prevent induction of learning-related cFos+ neurons in PreLC, but suppressed distributed network cFos induction, e.g. in vH (Fig. 6a). Therefore, together with local memory network reactivation, local cFos activity is necessary to induce distant memory network induction and time unit function.

To investigate the mechanisms through which cFos activity in local memory neurons might be necessary to induce distant memory network cFos expression upon local tagged neuron reactivation, we focused on the neurotrophin BDNF^11,12^. This growth factor has powerful roles in promoting learning-related plasticity^12,31,32^, and its presynaptic release depends on robust depolarization and calcium entry^12–14,33^. Furthermore, BDNF is required for long-lasting translation and synaptic plasticity during a 4–5 h time window after acquisition^32^. Indeed, local delivery of a specific small-molecule inhibitor of the BDNF receptor TrkB^34^ to PreLC prevented cFos expression specifically in PreLC upon reactivation of tagged vH cFos+ fear memory neurons (Fig. 6b). In parallel, and further supporting the notion that recruitment of the entire distributed memory network is required for binding, local delivery of the TrkB inhibitor suppressed memory binding in the vH-tagged neuron reactivation experiment (Fig. 6b). Furthermore, consistent with the notion that local BDNF signaling is critically important for time unit function, delivery of TrkB inhibitor to PreLC 1 h after cFC suppressed freezing to object at 7 h in the associational fear memory binding experiment (Supplementary Fig. 8).

To investigate whether BDNF might also be sufficient, together with network activity, for distant cFos+ neuron induction, we carried out BDNF delivery experiments. Indeed, local delivery of BDNF into PreLC rescued induction of cFos+ neurons specifically in PreLC upon reactivation of tagged vH cFos+ fear memory neurons when local vH cFos activity was inhibited (Fig. 6b). In control experiments, although BDNF by itself did induce some cFos+ neurons, it was not sufficient to induce an amount of cFos+ neurons comparable to fear memory-associated cFos+ neuron expression in PreLC in the absence of pharmacogenetic ligand to activate tagged vH cFos+ fear memory neurons (Supplementary Fig. 8). Together, these results are consistent with the notion that the activity of cFos in activated local memory neurons is critically important to mediate distant memory network recruitment through activity-dependent BDNF signaling in distant target regions.

## Discussion

We have shown that learning processes consist of dedicated 5 h time units, during which sufficient numbers of trials need to be carried out in order to produce learning, and individual shared learning-relevant elements are sufficient to combine otherwise unrelated behavioral experience, and to interfere with learning (Supplementary Fig. 9). Learning unit function depended critically on sustained competence by specific system-wide assemblies of cFos expressing neurons^22^, ensured through neuronal network activity, cFos protein expression and BDNF signaling in used brain structures.

Activity throughout brain structures used in the particular learning was critically important to maintain cFos expression and time unit function. Whether and in what way the ongoing distributed network activity was a specific consequence of learning remains to be determined. In principle, however, network activity might initiate spontaneously at any part of the network, e.g. as ripples or spindles^3,10,35^, and might then recruit and maintain system-wide networks in a cFos-dependent manner, e.g. through CREB function^7^, enhanced neuronal excitability and BDNF signaling. Notably, reactivating subsets of local cFos+ neurons was sufficient to recruit learning-specific system-wide assemblies for time unit function, providing a potential mechanism through which partial memory recall might open up a new time unit for further learning.

Our results are in good agreement with the notion that cell assemblies involved in learning and memory consist of interconnected neurons in multiple brain systems^8,9,22,23^. Our findings are further consistent with the notion that the 5 h time unit coincides with a time window of 5–6 h during which memories can be linked through a shared neural ensemble^6,7^. Accordingly, our findings suggest that facilitated memory ensemble merging during 5–6 h upon acquisition^6,7^ might be a key mechanism in learning, underlying integration of validated, related information to ensure learning, and preventing learning of spuriously or inconsistently linked experiences.

Our results provide evidence as to the mechanisms critically important to sustain time unit function during and beyond 5 h. We suggest that time unit function involves protracted processes of system-wide formation, active off-line maintenance and evidence-driven modification of cFos+ neuronal assemblies ensured through network-wide activity and coordinated throughout specific circuitry by cFos and BDNF activity (Supplementary Fig. 9). These notions are consistent and closely comparable to those of two recent studies on the linkage of memories during a limited time window after learning, and on the role of enhanced neuronal excitability in this process^6,7,36,37^. Our results further suggest that a major role of cFos expression induced upon learning is to implement time unit function through neuronal assembly maintenance, e.g. through enhanced neuronal excitability^6,7^. The endogenous mechanisms that limit time unit function to about 5 h remain to be determined, but negative regulation of CREB function is a candidate mechanism^7^. On the other hand, our findings do not exclude the possibility that plasticity induced during the 5 h time window, and involving cFos expression and function, might have additional roles in learning and memory, e.g. as a prerequisite for long-term memory consolidation^5,31^.

Our study uncovers remarkable implications of the dedicated 5 h time units on learning. This included a dramatic sensitivity to learning-related but contradicting information during a 5 h time window after initial learning. For example, robust fear conditioning to two different contexts within 5 h produced strongly reduced freezing to any of the two contexts upon recall. Interestingly, a previous study^7^ found that fear conditioning to different tones within 6 h could instead result in potentiation of freezing to the second tone upon recall, suggesting subtle distinctions between interference and synergy during time units for learning. In unfavorable cases, the high sensitivity to inconsistent associations might interfere with efficient learning. On the other hand, in addition to providing opportunities for validation in learning, a key advantage of the sensitivity to interference might involve preventing linkage of unrelated information in memory, a process that might majorly interfere with adaptive behavior and cognition. Future studies should define the molecular/cellular and circuit mechanisms that modify learning in the presence of inconsistent information, and their possible impairment in conditions affecting mental health.

That a single interfering episode was sufficient to modify learning, and that additional successful trials within the same time unit failed to rescue learning suggests that learning within the time unit does not resemble a process of quantitative evidence evaluation^38^. Instead, these observations suggest that, within the time unit, learning involves goal definition (i.e. learning content) through associative merging, mechanisms that link sufficient interactions with the task to thresholds for learning, and mechanisms that modify learning upon contradicting evidence. We also provide evidence that exhibiting learning-related improved performance requires the initiation of a new time unit. Accordingly, the time units for learning uncovered in this study might represent basic elements of a broader scheme to effectively and independently manage the identity of the learning content that enters memory networks in the brain.

## Methods

### Mice

PV-Cre and cFos-CreERT2 mice were from Jackson laboratories. Mice were kept in temperature-controlled rooms on a constant 12 h light–dark cycle. Before the behavioral experiment, mice were housed individually for 3–4 days and provided with food and water ad libitum. All animal procedures were approved and performed in accordance with the Veterinary Department of the Kanton of Basel-Stadt.

### Behavioral procedures

All behavioral experiments were carried out with male mice that were 60–75 days old at the onset of the experiment. For cFC, mice were placed in the training context (Habitest Unit, Coulbourn Instruments, Allentown, PA), were allowed to explore the apparatus for 3 min, and then received five foot shocks (1 s and 0.8 mA each, inter-trial interval: 30 s). The conditioning chamber was cleaned with 70% ethanol before and after each session, and specific odor (odor A, 2% acetic acid) was used to identify the conditioning context. Training context 1 (TR1) was rectangular and TR2 was cylindrical in shape. Identities of the contexts were maintained with the presence of two distinct odors, odor A (2% acetic acid: TR1) or B (0.25% benzaldehyde: TR2). Control mice were subjected to the same procedure without receiving foot shocks. We assessed contextual fear memory by returning mice to the training chamber after fear conditioning during 5 min, and analyzed freezing during a test period of 4 min (first minute excluded to avoid stress-related responses). Freezing was defined as complete absence of somatic mobility other than respiratory movements. For object binding, a falcon tube smeared with the odor corresponding to that used at conditioning or as otherwise specified was introduced during 30 min into the home cage. Freezing and avoidance behavior to the falcon tube were assessed on the next day for 5 min in a neutral context (no odor associated) that had been cleaned with water before and after testing.

In MWM training, a 140 cm pool filled with milky water was surrounded by three different objects placed as reference cues onto black curtains. A circular escape platform (10 cm diameter) was submerged 0.5 cm below the water surface. Mice were trained to find the platform during four trials a day or as otherwise specified, with inter-trial intervals of 5 min (or as otherwise specified) spent in their home cage. During training, mice were released from pseudo-randomly assigned start locations; they were allowed to swim for up to 60 s. At the end of each trial, mice were allowed to sit on the platform for 15 s; when trials were unsuccessful, mice were manually guided to the platform (only on the visible platform day, i.e. Day1). Performance was scored as latency to find the platform in each trial, and as the average of the four consecutive trials each day. Swim controls were age-matched mice, which were allowed to swim in the pool without escape platform, in a comparable training regime. For swim controls, trial durations for each day were adjusted to average values of training animals. Data were collected and analyzed using Viewer2 Software (Biobserve, Bonn, Germany).

For rotarod learning, mice were trained on an accelerating rotating rod (Ugo Basile srl; four trials per day and inter-trial intervals of 5 min in home cage, or as otherwise specified). A smooth scotch tape was used to enhance the level of difficulty. For each trial, mice were placed on the rod before the rotation was initiated, ensuring that they were able to sit on it for 5 s without falling. The training phase began only after the mice could successfully position themselves on the rod. Speed was increased in a step-wise fashion from 5 to 50 rpm over 5 min (maximal duration of each trial). Performance was scored as latency to fall in each trial, and as the average of the four consecutive trials each day. Activity controls were age-matched mice that were allowed to run on a rod at a fixed speed (10 rpm) in a comparable training regime.

In the FOR test, mice explored two objects (A/B) placed in an open arena for 10 min on day one, and returned to their home cage immediately after training. Next day, they were placed back into the original context for 5 min and tested for object recognition, when one of the two familiar objects had been replaced with a novel one (B/C). Discrimination indices were calculated as

(t*novel* – t*familiar*)/(t*novel* + t*familiar*). To avoid discrimination of the objects based on odor, both the arena and the objects were thoroughly wiped with 70% ethanol before and after each trial.

### Immunohistochemistry

Antibodies were as follows: rabbit anti-cFos (Santa Cruz biotechnology, sc-253, RRID: AB_2231996) 1:10,000; mouse anti-NeuN (Millipore, MAB377, AB_2298772), 1:1000; goat anti-PV (PVG-214, Swant biotechnologies, AB_10000345) 1:5000; α-Bungarotoxin, Alexa 488 Conjugate (Molecular Probes, Life Technologies, B-13422) 1:200. Secondary antibodies were Alexa Fluor 488 (Molecular Probes; A150077), 568 (Molecular Probes; A10037), or 647 (Molecular Probes; A31571, A21469); 1:500. dCA3 and vCA3 were analyzed at −1.82 to −1.94 mm and −2.80 to −3.16 mm from bregma, respectively; BLA was at −1.22 to −1.58 mm, M1 was at +1.58 to +1.8 mm from bregma and PreLC was at +1.8 to +1.98 mm from bregma. 3–5 sections per mouse were acquired and analyzed. The data per mouse was the average of the sections. Samples belonging to the same experiment (for example, from the mice of a given time point, with their respective controls) were acquired in parallel and with the same settings (laser power, 6%; master gain, 585 units, optical slice, 1 μm for cFos) on an LSM700 confocal microscope (Zeiss) using an EC Plan-Neofluar 40×/1.3 oil-immersion.

For c-Fos analysis, mice were perfused at the indicated time point after the training session, or as indicated (transcardially with 4% PFA in PBS, pH 7.4). Brains were kept in fixation solution overnight at 4 °C, then transferred to 30% sucrose solution for 24 h, sectioned (40 µm thickness) on a cryostat and stained while free-floating. The sections were blocked for 1 h at room temperature in 0.2% Triton-X100 in PBS and 10% BSA solution. The subsequent primary and secondary antibodies were diluted in 0.2% Triton-X100 in PBS and 3% BSA solution. The primary antibody incubation was overnight (~21 h) at 4 °C and the secondary antibody incubation was 2 h at room temperature, both with constant shaking. All c-Fos and NeuN immunopositive cells were quantified using an automatic spot-detection algorithm (Imaris 8.2.0, Bitplane AG; expected radius, 10 mm; quality level, 7), and their fraction expressed as a percentage of the total neuronal population. To adjust for variations in labeling intensity and microscope laser performance, the threshold for cFos+ cells was defined for each set of data (sections processed and analyzed in parallel) based on expected cage control values in vCA3 (2.5% cFos+/NeuN+ neurons) and dCA3 (1.5% cFos+/NeuN+ neurons). Briefly, the microscope settings were adjusted so that vCA3 and dCA3 values that had been consistently established by previous studies in our laboratory were matched, and the corresponding AU value was then used as a threshold to determine fractions of cFos+/NeuN+ neurons under various experimental conditions and across brain areas.

PV immunohistochemistry and intensity analysis was done as described^18^.

### Pharmacogenetics and pharmacology in vivo

All surgeries were conducted under aseptic conditions using a small animal stereotaxic instrument (David Kopf Instruments). Mice were anaesthetized with isoflurane (4% for induction, followed by 1.5–2.0%) in the stereotaxic frame during the entire surgery procedure and body temperature was maintained with a heating pad. Local virus delivery and drug treatments were carried out with a 33-gauge needle coupled to a 5 μl syringe (Hamilton, Reno, NV) or delivered using glass pipettes (tip diameter 10–20 μm) connected to a Picospritzer (Parker Hannifin Corporation). Coordinates relative to bregma were as follows: PreLC (anteroposterior (AP) + 2.0 mm, mediolateral (ML) + 0.5 mm, dorsoventral (DV, relative to dura) −2.1 mm and vH (AP −3.0 mm, ML + 2.9 mm, DV −3.5 mm). Drugs were injected at the rate of 100 nl/min to a final maximum volume of ~300 nl. After completion of injection the needle was left in its place for 5 min to allow for diffusion of the drug, and then slowly withdrawn. For virus injections, ~500 nl of the virus preparation was slowly injected using Picospritzer or Hamilton over a period of 10 min. After the end of the injection the pipette or needle was left in its place for further 10 min to allow for diffusion of the virus. All drugs and viruses were injected bilaterally.

For acute silencing, we delivered floxed PSAM-carrying AAV9 (excitation: rAAV9-CAG-flox-PSAM(Leu41Phe,Tyr116Phe)5HT3-WPRE) in PV-Cre mice. For activation or inhibition of c-Fos ensembles, floxed PSAM-carrying AAV9 (excitation: rAAV9-CAG-flox-PSAM(Leu41Phe,Tyr116Phe)5HT3-WPRE; or (inhibition: rAAV9-CBA-flox-PSAM(Leu141Phe,Tyr116Phe)GlyR-WPRE) were delivered locally in cFos-CreERT2 mice. To allow for transgene expression, mice were kept under home cage conditions for 10 days before any behavioral experiment. 4-Hydroxy tamoxifen (H6278, Sigma) dissolved in sunflower seed oil (Sigma) was injected i.p. at the dose of 50 mg/kg to activate Cre recombinase activity. PSAM agonist PSEM308 was injected i.p. at 5 mg/kg to activate PSAM channels.

Drugs used were as follows: T-5224 (1.5 µg/side, in 20% PVP and 10% DMSO, MedChemExpress, Inhibitor of cFos-AP1 transcription complex), MG-132 (100 µM, in 1% DMSO, Calbiochem); BDNF (0.5 µg/µl, in saline, 0.25 µg/side, Peprotech), ANA-12 (2 µg/µl, in 1% DMSO, 1 µg/side, Sigma, TrkB antagonist).

### Statistical analysis

Statistical analyses were performed using GraphPad Prism version 6.00 (GraphPad Software, La Jolla, CA, USA). As mentioned in the figure legends, depending on data-set, unpaired Student’s *t*-test, one-way ANOVA followed by Dunnet’s or Tukey posthoc test, two-way or repeated measures two-way ANOVA followed by Tukey or Sidak’s posthoc were performed; *P* < 0.05 in post hoc comparisons. Results are presented as mean ± s.e.m. All tests were two-tailed. Data distributions were assumed to be normal but not formally tested. The variance was comparable between groups for all metrics measured. The sample size per group (total number of animals collected over multiple repetitions of each experiment) is mentioned in the respective figure legends and was chosen and validated on the basis of previous studies performed in the laboratory. No statistical methods were applied to predetermine sample size. Male mice of closely comparable age were assigned randomly to experimental groups. Intensity analysis and freezing data were verified by investigators blinded to experimental conditions.

## Electronic supplementary material

Supplementary Information

## Data Availability

All relevant data are available from the authors.
