# Author Correction: Time units for learning involving maintenance of system-wide cFos expression in neuronal assemblies

**DOI:** 10.1038/s41467-019-11208-7

**Published:** 2019-07-12

**Authors:** Ananya Chowdhury, Pico Caroni

**Affiliations:** 1 Friedrich Miescher Institut, Maulbeerstrasse 66, CH-4058 Basel, Switzerland; 2 Present Address: Depts. of Neurobiology, Psychology, Psychiatry and Biobehavioral Sciences; Integrative Center for Learning and Memory;, Brain Research Institute, UCLA, Los Angeles, CA 90095 USA

**Keywords:** Cellular neuroscience, Consolidation

Correction to: *Nature Communications* 10.1038/s41467-018-06516-3, published online 8 October 2018.

The original version of this Article contained an error in the Acknowledgements, which incorrectly omitted from the end the following: ‘This project has received funding from the European Research Council (ERC) under the European Union’s Horizon 2020 research and innovation programme (MemoryDynamics - grant agreement No 694086).’ This has been corrected in both the PDF and HTML versions of the Article.

