# Supplementary material for: Time units for learning involving maintenance of system-wide cFos expression in neuronal assemblies
Source: Nat Commun. 2018 Oct 8;9:4122. doi: 10.1038/s41467-018-06516-3 (PMC6175937; doi:10.1038/s41467-018-06516-3)
Supplement: Supplementary file 1 — Supplementary Information [file 41467_2018_6516_MOESM1_ESM.pdf]

Supplementary information

Time units for learning involving maintenance of system-wide cFos expression in neuronal assemblies

Chowdhury and Caroni

Supplementary Figures

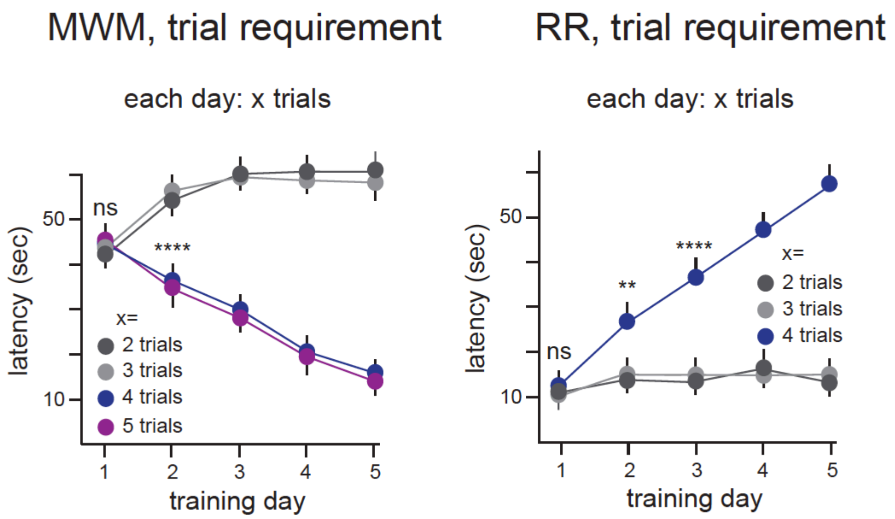

Chowdhury and Caroni Suppl. Fig.1

Supplementary Figure 1

Daily trial number requirements in water maze and rotarod learning under our experimental conditions. Learning curves as a function of daily trial numbers. Left: Morris water maze learning: Two-way RM ANOVA, Interaction, P<0.0001, n=5 mice each. Right: Rotarod learning: Two-way RM ANOVA, Interaction, P<0.0001, n=5 mice each. Error bars: SEM; p < 0.05 (*), 0.01 (**), 0.001 (***), 0.0001 (****). For a more detailed description of the statistical analysis please refer to Supplementary Table 7.

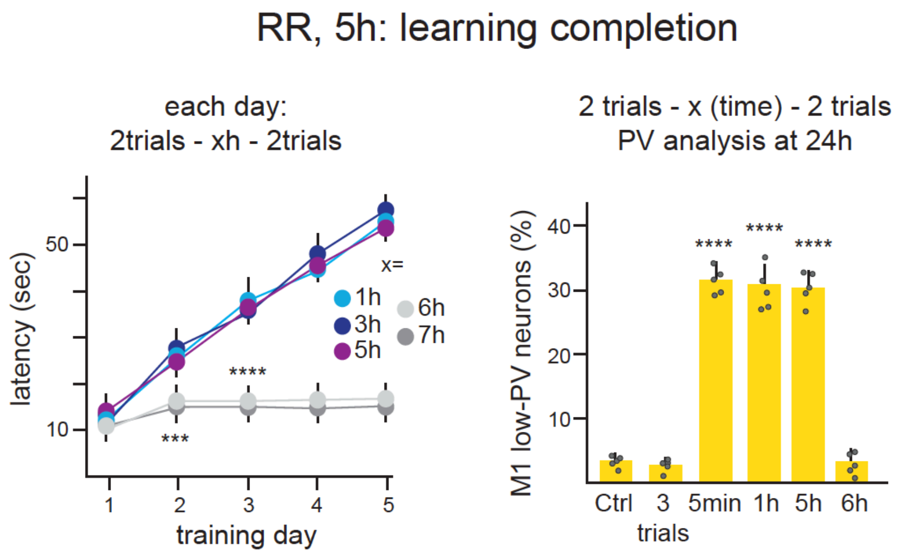

Chowdhury and Caroni Suppl. Fig.2

<u>Supplementary Figure 2</u>

Time unit for learning in rotarod training. x: numbers of hours between first and second group of 2 trials. Two-way RM ANOVA, Interaction, P<0.0001, n=5 each. Right: PV plasticity (low-PV contents in M1 at 24h): One-way ANOVA, P<0.0001, n=5 mice each. Error bars: SEM; p < 0.05 (*), 0.01 (**), 0.001 (***), 0.0001 (****). For a more detailed description of the statistical analysis please refer to Supplementary Table 8.

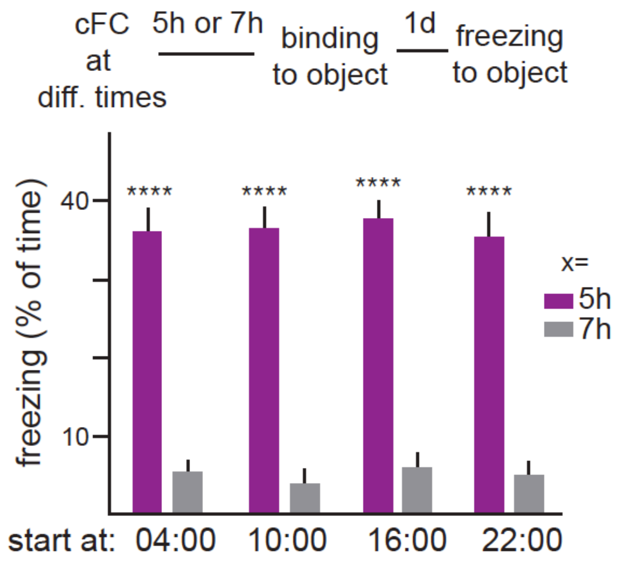

Chowdhury and Caroni Suppl. Fig.3

Supplementary Figure 3

The duration of the time unit for learning is not affected by the time of the day at which learning is initiated. Associational binding to fear memory; experimental conditions as in Fig. 1b. Two-way ANOVA, Interaction, P=0.8875, Interval of binding, P<0.0001, n=5 mice each. Error bars: SEM; p < 0.05 (*), 0.01 (**), 0.001 (***), 0.0001 (****). For a more detailed description of the statistical analysis please refer to Supplementary Table 9.

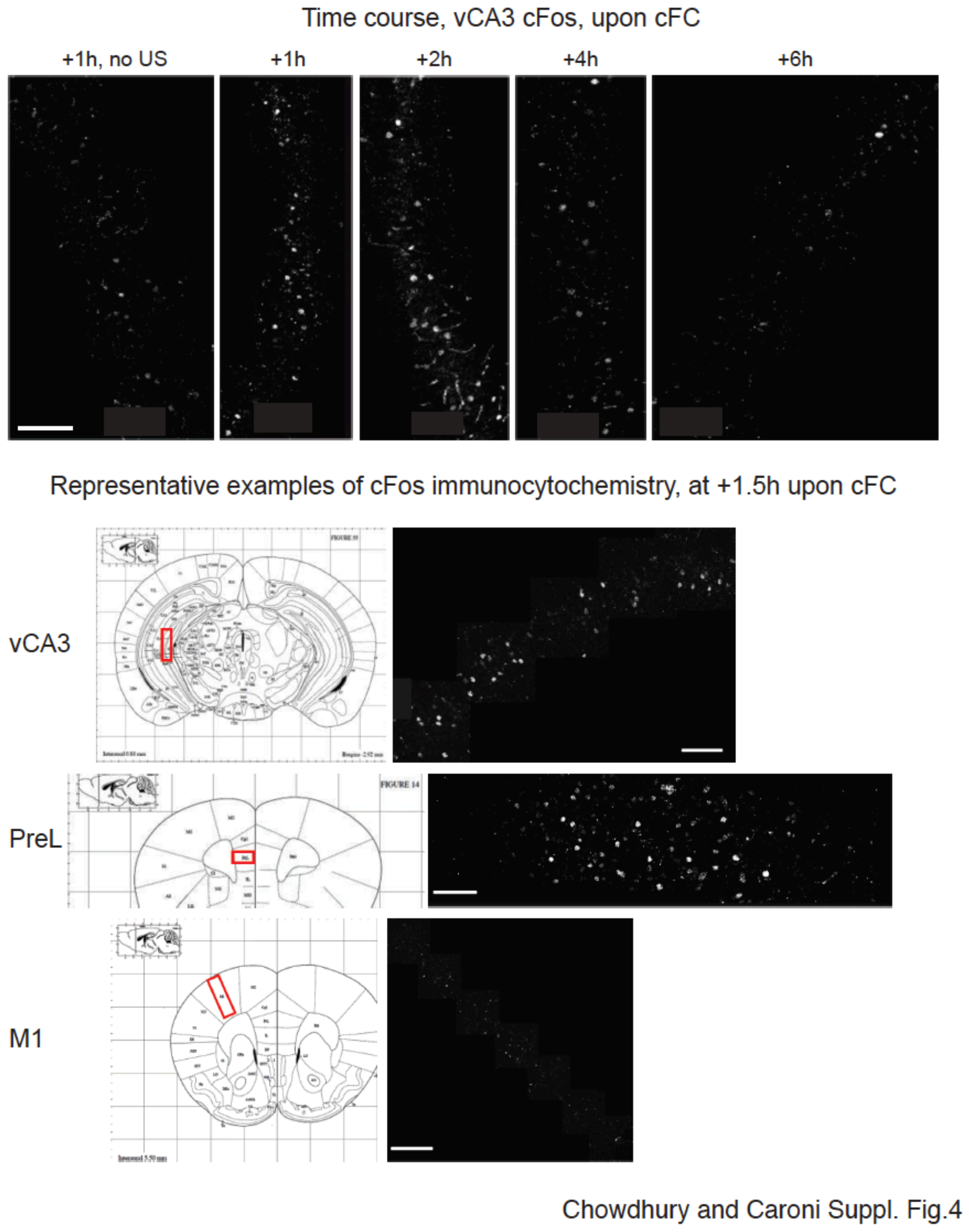

<u>Supplementary Figure 4</u>
cFos expression upon cFC.
Upper panels: Representative examples of time course data as shown in Fig. 3a; vH CA3.
Lower panels: Representative examples of cFos signals in vCA3, PreL, and M1 at +1.5h upon cFC. For orientation, corresponding images from The Mouse Brain in Stereotaxic Coordinates (Paxinos G, Franklin KBJ, Academic Press, 4th edition, 2012) are shown on the left.
Bars: 100 um.

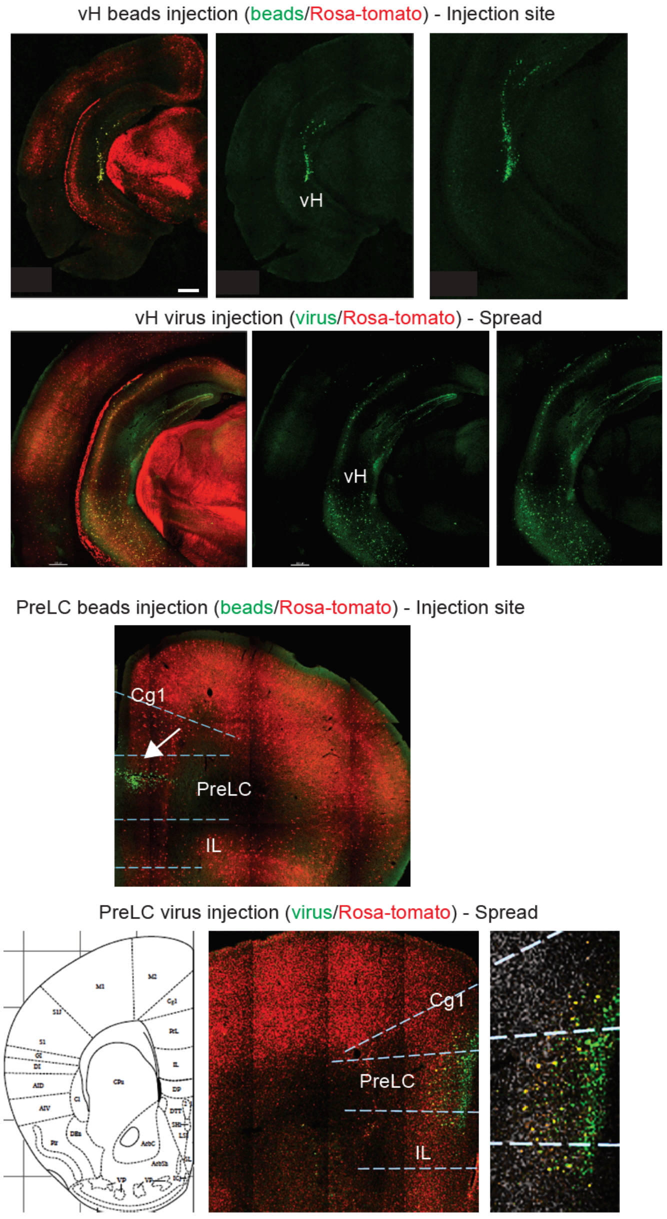

Chowdhury and Caroni Suppl. Fig.5

<u>Supplementary Figure 5</u>

Local delivery of AAV to vH and to PreLC. Representative examples of local treatment experiments as applied throughout the study. Beads: fluorescent beads to visualize injection site (arrow in PreLC); Virus: Bungarotoxin signal; Rosa-tomato: global histology marker. Right panels: higher magnification of images shown in left and center panels. For orientation, a corresponding image from The Mouse Brain in Stereotaxic Coordinates (Paxinos G, Franklin KBJ, Academic Press, 4th edition, 2012) is shown on the left of the panels on the bottom row. Bar: 500 um.

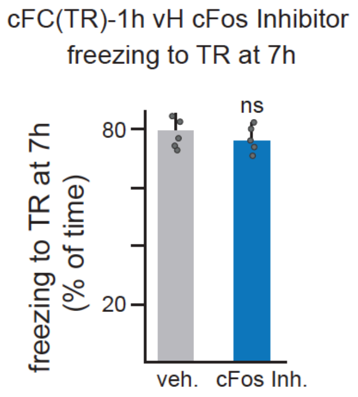

Chowdhury and Caroni Suppl. Fig.6

Supplementary Figure 6

Preventing binding to fear memory through vH inhibition at 1h does not interfere with fear memory to context as tested at 7h. n=5 mice each. Unpaired t-test, t(8)=0.5733, P=0.5822. Error bars: SEM; p < 0.05 (*), 0.01 (**), 0.001 (***), 0.0001 (****).

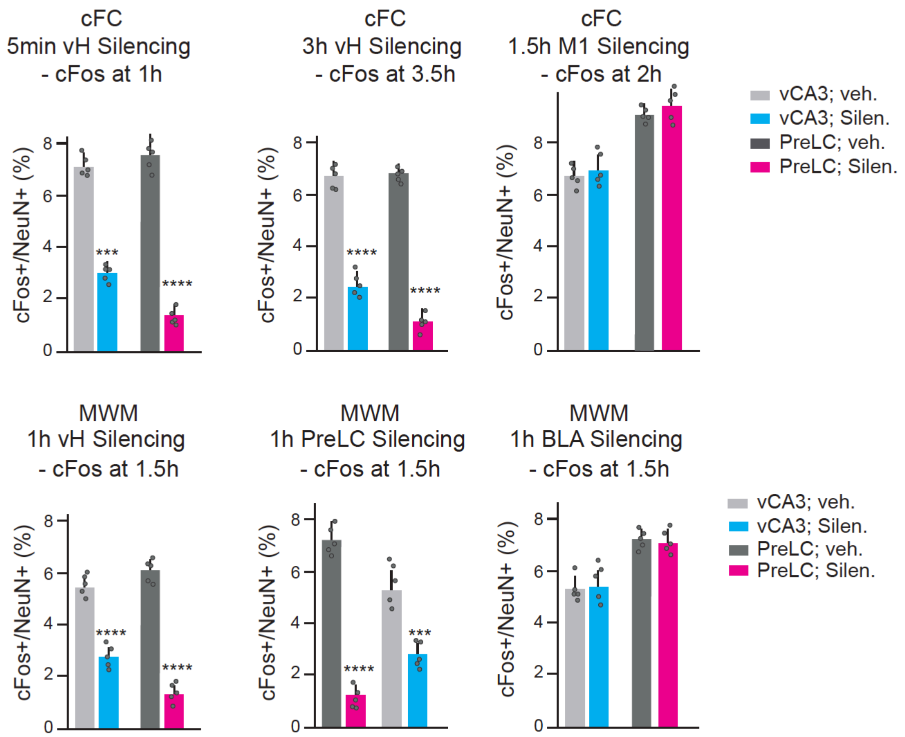

Chowdhury and Caroni Suppl. Fig.7

<u>Supplementary Figure 7</u>

Network activity for sustained cFos expression.

Upper panels: Silencing vH at 5min (left) [Two-way ANOVA, Interaction, P=0.0035, Treatment, P<0.0001, n=5 each] or 3h (center) upon cFC [Two-way ANOVA, Interaction, P=0.1283), Treatment, P<0.000, n=5 each] produces network-wide loss of cFos protein; in control experiments, silencing M1 at 1.5h (right) [Two-way ANOVA, Interaction, P=0.5546, Treatment, P=0.0494, n=5 each], does not affect cFos expression upon cFC.

Lower panels: Silencing vH [Two-way ANOVA, Interaction, P=0.0009, Treatment, P<0.0001, n=5 each] or PreLC [Two-way ANOVA, Interaction, P<0.0001, Treatment, P<0.0001, n=5 each] but not BLA [Two-way ANOVA, Interaction, P=0.5223, Treatment, P=0.4623, n=5 each], at 1h upon MWM learning (4 trials) produces network-wide loss of cFos protein at 1.5h.

Error bars: SEM; p < 0.05 (*), 0.01 (**), 0.001 (***), 0.0001 (****).

For a more detailed description of the statistical analysis please refer to Supplementary Table 10.

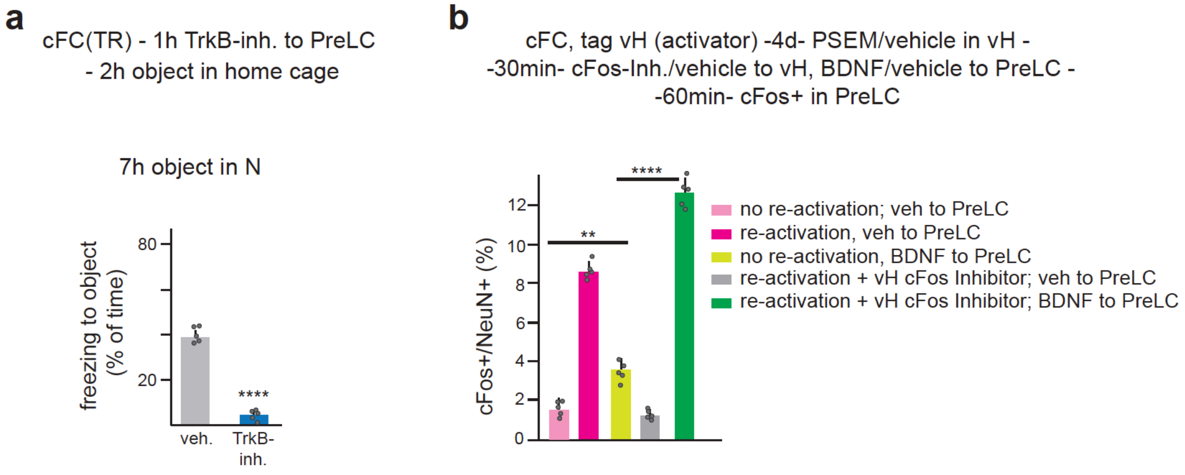

Chowdhury and Caroni Suppl. Fig.8

Supplementary Figure 8

Role of local BDNF signaling for time unit function and distant cFos induction.

(**a**) Local TrkB signaling during time unit for learning is critically important for time unit function, n=5 each. Unpaired t-test, $t(8)=10.97$, $P<0.0001$.

(**b**) cFos+ neuron contents in PreLC 1.5h after reactivation of tagged vH cFos+ neurons; vH cFos inhibitor and PreLC BDNF were delivered within 30min from tagged vH cFos+ neuron reactivation. Note how local delivery of BDNF to PreLC in the absence of tagged vH cFos+ neurons reactivation only induced a modest increase in cFos+ neuron contents in PreLC. One-way ANOVA, $P<0.0001$, n=5 each.

Error bars: SEM; $p <$ 0.05 (*), 0.01 (**), 0.001 (***), 0.0001 (****).

For a more detailed description of the statistical analysis please refer to Supplementary Table 11.

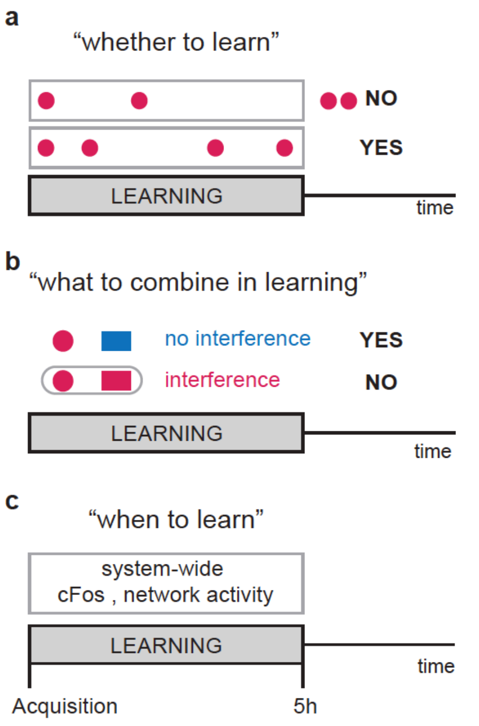

Chowdhury and Caroni Suppl. Fig. 9

Supplementary Figure 9

Time units for learning involving maintenance of system-wide cFos expression in neuronal assemblies.

(**a**) Within 5h time units for learning (gray box) sufficient trials need to be executed (red circles) to determine whether learning occurs (no/yes).

(**b**) Within the time units, learning-related shared features are associated to determine what to learn; associations (red circle/red square), if leading to contradictions, can produce interference, disrupting learning within the time unit.

(**c**) Induction (acquisition) and maintenance of network activity and cFos expression in distributed brain systems involved in the particular type of learning determine when to learn.

Supplementary Table 1
Additional information to statistical analysis in Fig. 1.

Figure Number	Statistics test	No of mice	Degrees of freedom (F)	P-Value	Post hoc test
1a	Two-way RM ANOVA	5,8,5,5,5	F(4,23)=11.1 (Interaction)<br>F(4,23)=15.56 (Time intervals)<br>F(1,23)=35.87 (Day)	p<0.0001<br>p<0.0001<br>p<0.0001	Tukey posthoc: day1: between groups-ns, day2: 6h or 7h vs 1h or 3h or 5h-p<0.0001
1a	One-way ANOVA	7,5,11,5,5	F(4,28)=105.6	p<0.0001	Dunnett's post hoc: compared to Ctrl, 3trial-ns; 5min and 3h-p <0.0001 and 6h-ns
1b	One-way ANOVA	5,9,5,5,5,5,5,5	F(7,36)=104.9	p<0.0001	Dunnett's post hoc: compared to no object, 3h object no odor-ns; 3h object odor B-ns; 1h, 3h and 5h-p<0.0001; 6h-ns; 7h- ns;
1b	Unpaired t-test	12,9	t(19)=0.8568	p=0.4022	-

Supplementary Table 2
Additional information to statistical analysis in Fig. 2.

Figure Number	Statistics test	No of mice	Degrees of freedom (F)	P-Value	Post hoc test
2a	Unpaired t-test	5,5	t(8)=30.14	p<0.0001	-
2a	Two-way ANOVA	5,5,6,6	F(1,18)=0.1512 (Interaction)<br>F (1, 18) = 152.7 (Time intervals)<br>F (1, 18) = 0.235 (Context	p=0.7020<br>p<0.0001<br>p=0.6337	Sidak's post hoc: TR1 3h vs 6h-p<0.0001, TR2 3h vs 6h-p<0.0001
2b	Two-way RM ANOVA		F(1,8)=9.801 (Interaction)<br>F(1,8)=37.17	p=0.0140<br>p=0.0003	Sidak's post hoc: day1 3h vs 7h-ns,

			(Time intervals) F(1,8)=1.918 (Day)	p=0.2035	day2 3h vs 7h - p=0.0001
2b	Unpaired t-test	5,5	t(8)=9.272	p<0.0001	-
2c	Two-way RM ANOVA	5,5,5	F(2,12)=8.833 (Interaction) F(2,12)=21.43 (Condition) F(1,12)=11.12 (Day)	p=0.0044 p=0.0001 p=0.0059	Tukey post hoc: day1 between groups- ns, day2 no FOR vs FOR no match- ns, no FOR vs FOR match- p<0.0001, FOR no match vs FOR match- p<0.0001
2c	One-way ANOVA	5,5,5	F(2,12)=14.28	p=0.0007	Tukey post hoc: no MWM vs MWM no match -ns, no MWM vs MWM match, p=0.0020, MWM no match vs MWM match, p=0.0012
2c	Two-way RM ANOVA	5,5,5	F(2,12)=6.976 (Interaction) F(2,12)=34.1 (Condition) F(1,12)=16.28 (Day)	p=0.0098 p<0.0001 p=0.0017	Tukey post hoc: day1 between groups, ns, day2 no FOR vs FOR no match p=0.9090, ns, no FOR vs FOR match, p<0.0001, FOR no match vs FOR match, p<0.0001
2c	One-way ANOVA	5,5,5	F(2,12)=13.38	p=0.0009	Tukey post hoc: no MWM vs MWM no match -ns, no MWM vs MWM match, p=0.0026, MWM no match vs MWM match, p=0.0016

2d	Two-way RM ANOVA	5,5	F(1,8)=15.56 (Interaction)<br>F(1,8)=12.21 (Time interval)<br>F(1,8)=6.589 (Day)	p=0.0043<br>p=0.0081<br>p=0.0333	Sidak's post hoc: day1 4h vs 7h - ns,<br>day2 4h vs 7h- p=0.0002
2e	Two-way RM ANOVA	5,5	F(1,8)=7.819 (Interaction)<br>F(1,8)=6.664 (Time interval)<br>F(1,8)=11.5 (Day)	p=0.0233<br>p=0.0325<br>p=0.0095	Sidak's post hoc, day1 4h vs 7h-ns, day2 4h vs 7h- p=0.0031

<u>Supplementary Table 3</u>
Additional information to statistical analysis in Fig. 3.

Figure Number	Statistics test	No of mice	Degrees of freedom (F)	P-Value	Post hoc test
3a cFC	One-way ANOVA	5	vCA3: F (6, 28) = 50.47<br>dCA3: F (6, 28) = 30.74<br>PreLC: F (6, 28) = 51.3<br>BLA: F (6, 28) = 195.4<br>M1: F (6, 28) = 0.8437	p<0.0001<br>p<0.0001<br>p<0.0001<br>p<0.0001<br>p=0.5472	Dunnett's post hoc: Compared each time point (1-6h) with the corresponding 0h (baseline) of that area.<br>The time points 1h, 2h, 3h, 4h (compared with corresponding 0h) of vCA3, dCA3, PreLC and BLA: p<0.0001. The 5h time point of vCA3: p=0.0008, dCA3: p=0.0004, PreL and BLA: p=0.0002.<br>The 6h time point of vCA3, dCA3, PreLC, BLA: ns.<br>For M1, every time point: ns
3a MWM	One-way ANOVA	5	vCA3: F (4, 20) = 51.28<br>dCA3: F (4, 20) = 21.27	p<0.0001<br>p<0.0001	Dunnett's post hoc: Compared each time point (1-6h) with the

			PreLC: F (4, 20) = 49.05<br>BLA: F (4, 20) = 0.4278<br>M1: F (4, 20) = 0.03252	p<0.0001<br><br>p=0.7868<br><br>p=0.9978	corresponding 0h (baseline) of that area.<br>The time points 1h, 2h of vCA3, dCA3 and PreLC: p<0.0001. The 4h time point of vCA3: p= 0.0002, dCA3: p= 0.0049; PreL: p<0.0001. The 6h time point of vCA3, dCA3, PreLC: ns. For BLA and M1, every time point: ns
3b cFC	Two-way ANOVA	5,5,5,5	F (1, 16) = 0.8659 (Interaction)<br>F (1, 16) = 0.02369 (area)<br>F (1, 16) = 108 (Treatment)	p=0.3659<br><br>p=0.8796<br><br>p<0.0001	Sidak's post hoc: For both vH inj. and PreL inj.<br>veh vs cfos Inh: p<0.0001
3b MWM	Two-way ANOVA	5,5,5,5	F (1, 16) = 0.636 (Interaction)<br>F (1, 16) = 0.002532 (area)<br>F (1, 16) = 69.74 (Treatment)	p=0.4368<br><br>p=0.9605<br><br>p<0.0001	Sidak's post hoc: For vH inj,<br>veh vs cfos Inh : p=0.0001<br>For PreL inj,<br>veh vs cfos Inh : p<0.0001
3c	Two-way ANOVA	5,5,5,5,5,5	F (2,24) = 12.34 (Interaction)<br>F (2,24) = 3.383 (Brain area analyzed)<br>F (1, 16) = 433.5 (Treatment)	p=0.0002<br><br>p=0.0508<br><br>P<0.0001	Sidak's post hoc: For vCA3, PreL and BLA<br>veh vs silencing: p<0.0001
3c	Two-way ANOVA	5,5,5,5,5,5	F (2, 24) = 9.595 (Interaction)<br>F (2,24) = 0.5061 (Brain area analyzed)<br>F (1, 16) = 390 (Treatment)	p=0.0009<br><br>p=0.6091<br><br>p<0.0001	Sidak's post hoc: For vCA3, PreL and BLA<br>veh vs silencing: p<0.0001

3d	Two-way ANOVA	5,5,5,5	F (1, 16) = 0.1107 (Interaction)<br>F (1, 16) = 0.001718 (Brain area injected)<br>F (1, 16) = 353.5 (Treatment)	p=0.7436<br>p=0.9674<br>p<0.0001	Sidak's post hoc: For both vH inj. and PreL inj veh vs silencing: p<0.0001
3d	Two-way ANOVA	5,5,5,5	F (1, 16) = 1.289 (Interaction)<br>F (1, 16) = 0.04866 (Brain area injected)<br>F (1, 16) = 38.36 (Treatment)	p=0.2730<br>p=0.8282<br>p<0.0001	Sidak's post hoc: For vH inj. veh vs silencing: p=0.005, For PreL inj. veh vs silencing: p=0.0002

<u>Supplementary Table 4</u>
Additional information to statistical analysis in Fig. 4.

Figure Number	Statistics test	No of mice	Degrees of freedom (F)	P-Value	Post hoc test
4a	vCA3: Two-way ANOVA	9,9,5,6,5,6	F(2,34)=24.22 (Interaction)<br>F(1,34)=70.15 (Treatment)<br>F(2,34)=21.96 (Time points)	p<0.0001<br>p<0.0001<br>p<0.0001	Sidak's post-hoc: veh vs MG132, at 7h and 9h- p<0.0001, at 11h, p=0.8569, ns
	PreLC: Two-way ANOVA		F(2,34)=104 (Interaction)<br>F(1,34)=448.9 (Treatment)<br>F(2,34)=98.97 (Time points)	p<0.0001<br>p<0.0001<br>p<0.0001	Sidak's post-hoc: veh vs MG132, at 7h and 9h- p<0.0001, at 11h, p=0.9865, ns;
	BLA: Two-way ANOVA		F(2,34)=20.11 (Interaction)<br>F(1,34)=81.41 (Treatment)<br>F(2,34)=23.72 (Time points)	p<0.0001<br>p<0.0001<br>p<0.0001	Sidak's post-hoc: veh vs MG132, at 7h and 9h - p<0.0001, at 11h, p=0.9997, ns
4a	PreLC: Two-way ANOVA	5,5,5,5,5,5	F(2,24)=86.4 (Interaction)<br>F(1,24)=282.2	p<0.0001<br>p<0.0001	Sidak's post-hoc: veh vs MG132, at 7h and 9h -

			(Treatment)<br>F(2,24)=76.86 (Time points)	 p<0.0001	p<0.0001, at 11h, p=0.7335, ns;
	vCA3: Two-way ANOVA		F(2,24)=46.77 (Interaction)<br>F(1,24)=168.6 (Treatment)<br>F(2,24)=46.83 (Time points)	p<0.0001<br><br>p<0.0001<br><br>p<0.0001	Sidak's post-hoc: veh vs MG132, at 7h and 9h - p<0.0001, at 11h, p=0.9739, ns;
	BLA: Two-way ANOVA		F(2,24)=96.92 (Interaction)<br>F(1,24)=368.7 (Treatment)<br>F(2,24)=95.51 (Time points)	p<0.0001<br><br>p<0.0001<br><br>p<0.0001	Sidak's post-hoc: veh vs MG132, at 7h and 9h - p<0.0001, at 11h, p= 0.9897, ns;
4b	vH treatment: Two-way ANOVA	5,5,5,5,5,5	F (2,24) = 48.09 (Interaction)<br>F (2,24) = 44.12 (Time of binding)<br>F (1, 24) = 166.2 (Treatment)	p<0.0001<br><br>p<0.0001<br><br>p<0.0001	Sidak's post-hoc: veh vs MG132, at 7h and 9h- p<0.0001, at 11h, p=0.9349
4b	PreL treatment: Two-way ANOVA	5,5,5,5,5,5	F (2,24) = 44.71 (Interaction)<br>F (2,24) = 47.9 (Time of binding)<br>F (1, 24) = 145.3 (Treatment)	p<0.0001<br><br>p<0.0001<br><br>p<0.0001	Sidak's post-hoc: veh vs MG132, at 7h and 9h- p<0.0001, at 11h, p=0.8907,
4b	Two-way ANOVA	5,5,5,5	F (1,16) = 0.4457 (Interaction)<br>F (1,16) = 0.004367 (Time point)<br>F (1, 16) = 231.7 (Treatment)	p=0.5139<br><br>p=0.9481<br><br>p<0.0001	Sidak's post-hoc: veh vs cFos inh, both 3.5h and 6.5h- p<0.0001
4b	Two-way RM ANOVA	5,5	F(1,8)=19.42 (Interaction)<br>F(1,8)=22.55 (Treatment)<br>F(1,8)=1.767 (Day)	p=0.0023<br><br>p=0.0014<br><br>p=0.2204	Sidak's post-hoc: veh vs MG132, day1, p= 0.9494, ns, day2, p<0.0001

4b	Two-way RM ANOVA	5,5	F(1,8)=35.62 (Interaction)<br>F(1,8)=52.14 (Treatment)<br>F(1,8)=2.463 (Day,	p=0.0003<br><br>p<0.0001<br><br>p=0.1552	Sidak's post-hoc: veh vs MG132, day1, p=0.8403, ns, day2, p<0.0001

Supplementary Table 5
Additional information to statistical analysis in Fig. 5.

Figure Number	Statistics test	No of mice	Degrees of freedom (F)	P-Value	Post hoc test
5d	Two-way ANOVA	5,6,6,6, 5,5	F (2,27) = 0.0004625 (Interaction)<br>F (1, 27) = 0.5777 (Time of binding)<br>F (2,27) = 193.6 (Condition)	p=0.9995<br><br>p=0.4538<br><br>p<0.0001	Sidak's post hoc: at 1h and 3h, veh cFC vs PSEM cFC- p<0.0001; PSEM MWM vs PSEM cFC- p<0.0001; veh cFC vs PSEM MWM-ns
5d	Two-way RM ANOVA	5,5,5,5	F(3,16)=8.019 (Interaction)<br>F(3,16)=7.098 (Condition)<br>F(1,16)=0.3159 (Day)	p=0.0017<br><br>p=0.0030<br><br>p=0.5819	Sidak's post hoc: day1 between groups- ns,<br>day2 veh MWM vs PSEM MWM, p<0.0001, veh MWM vs PSEM cFC, p=0.9850, veh MWM vs PSEM no 2trial- ns
5d	One-way ANOVA	5,5,5	F(2,12)=38.81	p<0.0001	Dunnett's post hoc: veh cFC vs PSEM cFC, p< 0.0001, veh cFC vs PSEM MWM-ns

Supplementary Table 6
Additional information to statistical analysis in Fig. 6.

Figure Number	Statistics test	No of mice	Degrees of freedom (F)	P-Value	Post hoc test
6a	One-way ANOVA	5,5,5,5	F(3,16)=207.7	p<0.0001	Dunnett's post hoc: Compared to vCA3, dCA3, PreL and BLA- p< 0.0001
6a	Unpaired t-test	5,6	t(9)=10.17,	p<0.0001	-
6a	One-way ANOVA	5,5,5,5	F(3,16)=289.3	p<0.0001	Dunnett's post hoc: Compared to PreL, vCA3, dCA3 and BLA-p< 0.0001
6b	One-way ANOVA	5,5,5,5	F(3,16)=139.7	p<0.0001	Dunnett's post hoc: vCA3 vs dCA3, p=0.9999, ns, vCA3 vs PreL, p< 0.0001 and vCA3 vs BLA, p=0.9999, ns
6b	Unpaired t-test	5,5	t(8)=8.525	p<0.0001	-
6b	One-way ANOVA	5,5,5,5	F(3,16)=199.2	p<0.0001	Dunnett's post hoc: vCA3 vs dCA3, p< 0.0001, vCA3 vs PreL,p=0.8044,ns and vCA3 vs BLA, p< 0.0001

Supplementary Table 7
Additional information to statistical analysis in Supplementary Figure 1.

Figure Number	Statistics test	No of mice	Degrees of freedom (F)	P-Value	Post hoc test
MWM	Two-way RM ANOVA	5 each	F(12,64)=27.94 (Interaction) F(4,64)=16.72 (Day) F(3,16)=180.5 (Trial number)	p<0.0001 p<0.0001 p<0.0001	Sidak's post-hoc: day1 between groups-ns, from day2 onwards, 2trials, 3trials vs 4trials, 5trials, p<0.0001
RR	Two-way RM ANOVA	5 each	F(8,48)=22.95 (Interaction) F(4,48)=56.43 (Day) F(2,12)=184 (Trial number)	p<0.0001 p<0.0001 p<0.0001	Sidak's post-hoc: day1 between groups-ns, from day2, 3trials vs 4trials, p=0.0023, day3 onwards, p<0.0001

Supplementary Table 8
Additional information to statistical analysis in Supplementary Figure 2.

Figure Number	Statistics test	No of mice	Degrees of freedom (F)	P-Value	Post hoc test
RR	Two-way RM ANOVA	5 each	F(16,80)=32.46 (Interaction) F(4,80)=276.7 (Day) F(4,20)=155 (Time interval)	p<0.0001 p<0.0001 p<0.0001	Sidak's post-hoc: day1 between groups-ns, from day2, 5h vs 7h, p=0.0003, day3 onwards, 1h, 3h, 5h vs 6h, 7h, p<0.0001
PV	One-way ANOVA	5 each	F(5,24)=120.8	p<0.0001	Dunnett's post hoc: compared to Ctrl, 3trial p=0.9690, ns, 5min, 1h and 5h, p <0.0001 and 6h, p= 0.8113, ns.

Supplementary Table 9
Additional information to statistical analysis in Supplementary Figure 3.

Figure Number	Statistics test	No of mice	Degrees of freedom (F)	P-Value	Post hoc test
3	Two-way ANOVA	5 each	$F(3, 32) = 0.2118$ (Interaction)<br>$F(1, 32) = 536.8$ (Interval of binding)<br>$F(3, 32) = 0.6837$ (Time of day)	$p=0.8875$<br>$p<0.0001$<br>$p=0.5685$	Sidak's post hoc: 5h vs 7h, irrespective of time of day, $p<0.0001$

Supplementary Table 10
Additional information to statistical analysis in Supplementary Figure 7.

Figure Number	Statistics test	No of mice	Degrees of freedom (F)	P-Value	Post hoc test
cFC 5min vH silencing	Two-way ANOVA	5 each	$F(1, 16) = 11.7$ (Interaction)<br>$F(1, 16) = 0.1772$ (Brain area analyzed)<br>$F(1, 16) = 112.8$ (Treatment)	$p=0.0035$<br>$p=0.6794$<br>$p<0.0001$	Sidak's post hoc: veh vs silenced, vCA3, $p=0.0002$, PreL, $p<0.0001$
cFC 3h vH silencing	Two-way ANOVA	5 each	$F(1, 16) = 2.573$ (Interaction)<br>$F(1, 16) = 2.251$ (Brain area analyzed)<br>$F(1, 16) = 113$ (Treatment)	$p=0.1283$<br>$p=0.1530$<br>$p<0.0001$	Sidak's post hoc: veh vs silenced, vCA3 and PreL, $p<0.0001$
cFC 1.5h M1 silencing	Two-way ANOVA	5 each	$F(1, 16) = 0.3643$ (Interaction)	$p=0.5546$<br>$p=0.0008$	Sidak's post hoc: veh vs silenced, vCA3, $p=0.1378$, PreL, $p=0.5065$

			F (1, 16) = 17.02 (Brain area analyzed) F (1, 16) = 4.523 (Treatment)	p=0.0494	
MWM vH silencing	Two-way ANOVA	5 each	F (1, 16) = 16.53 (Interaction) F (1, 16) = 0.5828 (Brain area analyzed) F (1, 16) = 237.4 (Treatment)	p=0.0009 p=0.4563 p<0.0001	Sidak's post hoc: veh vs silenced, vCA3 and PreL, p<0.0001
MWM PreL silencing	Two-way ANOVA	5 each	F (1, 16) = 44.95 (Interaction) F (1, 16) = 0.02129 (Brain area analyzed) F (1, 16) = 171.9 (Treatment)	p<0.0001 p=0.8858 p<0.0001	Sidak's post hoc, veh vs silenced, vCA3, p=0.0007, PreL, p<0.0001
MWM BLA silencing	Two-way ANOVA	5 each	F (1, 16) = 0.428 (Interaction) F (1, 16) = 7.647 (Brain area analyzed) F (1, 16) = 0.5672 (Treatment)	p=0.5223 p=0.0138 p=0.4623	Sidak's post hoc: veh vs silenced, vCA3, p=0.9970, PreL, p=0.5571

Supplementary Table 11
Additional information to statistical analysis in Supplementary Figure 8.

Figure Number	Statistics test	No of mice	Degrees of freedom (F)	P-Value	Post hoc test
8a	Unpaired t-test	5,5	$t(8)=10.97$	$p<0.0001$	-
8b	One-way ANOVA	5 each	$F(4,20)=122.9$	$p<0.0001$	Tukey's post hoc: compared to no re-activation+ veh to PreL (Ctrl), no re-activation+ BDNF to PreL, p=0.0088; compared to no re-activation+ BDNF to PreL, re-activation + cFos Inh to vH+ BDNF to PreL, p<0.0001
